# Causes of death among early-onset colorectal cancer population in the United States: a large population-based study

**DOI:** 10.3389/fonc.2023.1094493

**Published:** 2023-04-24

**Authors:** Yuerong Chen, Lanping He, Xiu Lu, Yuqun Tang, Guanshui Luo, Yuji Chen, Chaosheng Wu, Qihua Liang, Xiuhong Xu

**Affiliations:** ^1^ Minimally Invasive Tumor Therapies Center, Guangdong Second Provincial General Hospital, Guangzhou, China; ^2^ Department of Gastroenterology, Fogang County People’s Hospital, Fogang, China; ^3^ Center of Digestive Endoscopology, The Second People’s Hospital of Luoding City, Luoding, China; ^4^ Department of Acupuncture and Massage Rehabilitation, Integrated Hospital of Traditional Chinese Medicine, Southern Medical University, Guangzhou, China

**Keywords:** early-onset colorectal cancer, cause of death, prognostic factor, surveillance, survivorship

## Abstract

**Background:**

Early-onset colorectal cancer (EOCRC) has an alarmingly increasing trend and arouses increasing attention. Causes of death in EOCRC population remain unclear.

**Methods:**

Data of EOCRC patients (1975–2018) were extracted from the Surveillance, Epidemiology, and End Results database. Distribution of death was calculated, and death risk of each cause was compared with the general population by calculating standard mortality ratios (SMRs) at different follow-up time. Univariate and multivariate Cox regression models were utilized to identify independent prognostic factors for overall survival (OS).

**Results:**

The study included 36,013 patients, among whom 9,998 (27.7%) patients died of colorectal cancer (CRC) and 6,305 (17.5%) patients died of non-CRC causes. CRC death accounted for a high proportion of 74.8%–90.7% death cases within 10 years, while non-CRC death (especially cardiocerebrovascular disease death) was the major cause of death after 10 years. Non-cancer death had the highest SMR in EOCRC population within the first year after cancer diagnosis. Kidney disease [SMR = 2.10; 95% confidence interval (CI), 1.65–2.64] and infection (SMR = 1.92; 95% CI, 1.48–2.46) were two high-risk causes of death. Age at diagnosis, race, sex, year of diagnosis, grade, SEER stage, and surgery were independent prognostic factors for OS.

**Conclusion:**

Most of EOCRC patients died of CRC within 10-year follow-up, while most of patients died of non-CRC causes after 10 years. Within the first year after cancer diagnosis, patients had high non-CRC death risk compared to the general population. Our findings help to guide risk monitoring and management for US EOCRC patients.

## Introduction

Colorectal cancer (CRC) is the third most common cancer and the second leading cause of cancer death worldwide, with more than 1.9 million new cases and 935,000 deaths in 2020 (1). The incidence and mortality rate of CRC have declined in the last several decades (2), whereas the incidence of CRC in population < 50 years (deemed early-onset colorectal cancer, EOCRC) is increasing alarmingly (3). It is reported that CRC is a leading cause of cancer incidence and death in those aged 20–49 in the United States (4).

Increasing incidence and mortality rate of EOCRC require more understanding and action to this disease. With the advancement of cancer treatment, CRC patients, especially those young, have longer survival expectancy. Analyzing distribution of causes of death helps to guide high-risk disease monitoring and prevention in EOCRC survivors, which plays an important role in improving overall survival. Some previous studies analyzed causes of death in CRC patients with all age (5–7) or old age (8, 9). However, to the best of our knowledge, few recent studies focus on causes of death in EOCRC population. Evidence from previous studies indicated that EOCRC is different from average-onset CRC (AOCRC) both in clinical characteristics and pathogenesis (10), and it should be distinct from traditional CRC (11). Compared to AOCRC patients, EOCRC patients have less comorbidities and better basic condition, but they receive more aggressive anti-cancer treatment (12, 13), which may result in increased long-term non-cancer death risk. Therefore, we hypothesize that distribution of causes of death in EOCRC population is different from that of AOCRC.

To identify high-risk diseases threatening EOCRC survivors’ life and guide surveillance, we analyzed causes of death in EOCRC population using a national cancer database with long-term follow-up. Furthermore, the risk of each cause of death is compared with the general population in the USA.

## Methods

### Data source

Data of the present study were obtained from the Surveillance, Epidemiology and End Results (SEER) database using SEER*Stat software (version 8.4.0) after submitting the data use agreement and getting permission. The SEER database, which covers approximately 34.6% population in the United States, is the authoritative program of the National Cancer Institute (NCI) (14). This national large-scale data with long-term follow-up information is an ideal source for causes of death analysis. Patients’ informed consents and Institutional Review Board (IRB) approval are not required, since these data are publicly available in the database.

### Study design and participants

This is a national population-based retrospective study with long-term follow-up. To delineate the rough trends of EOCRC mortality in the last several decades, we first performed the joinpoint analysis in the US general population using data from the Centers for Disease Control and Prevention Wide-Ranging Online Data for Epidemiologic Research (CDC WONDER, 1999–2019) (15). Distribution of causes of death in EOCRC population was analyzed at different follow-up times to primarily identify high-risk diseases. Standard mortality ratios (SMRs) and absolute excess risks (AERs) of each cause of death in the overall EOCRC population and subgroups were calculated to compare death risk with the US general population. Furthermore, univariate and multivariate analyses were conducted to identify independent prognostic factors correlated with patients’ survival. Patients who survived 0–1 months after EOCRC diagnosis were excluded for causes of death analysis due to very short survival time. The report of the present study follows the STROBE guidelines (16).

Patients in the SEER database who met the following eligibility criteria were included: (1) histological confirmation of colon or rectal cancer as the primary cancer, (2) age at diagnosis between 20 and 49 years old, and (3) diagnosed between 1975 and 2018. Patient exclusion criteria were as follows: (1) death certification or autopsy only, (2) unknown causes of death or race, and (3) without active follow-up.

### Variables and outcomes

Patient variables include age, race, sex, year of diagnosis, grade, SEER stage, surgery, survival months, and cause of death. Patient age was categorized as 20–29, 30–39, and 40–49. Races included black, white, others, and unknown. Year of diagnosis was classified into four groups: 1975–1984, 1985–1994, 1995–2004, and 2005–2018. Tumor grade was categorized as grade I–IV, others, and unknown. SEER stage included localized, regional, distant, and unknown. Surgery was classified as yes, no, and unknown. Cause of death recode in the SEER database is based on the International Classification of Diseases (ICD) versions 8–10 since 1969 (17). Non-cancer causes of death in this study were further categorized as follows: cardiocerebrovascular diseases, infection, diabetes mellitus, Alzheimer’s disease, respiratory diseases, digestive diseases, kidney diseases, suicide, accidents and homicide, and other non-neoplastic diseases (**Supplementary Table S1**).

In this study, the primary end points for prognostic factor analysis were overall survival (OS), which was defined as the duration from cancer diagnosis to death from any causes. Patients who were still alive at the last follow-up time (31 December 2018) were viewed as censored events.

### Statistical analysis

Chi-square test was used to compare patient baseline characteristics. Trends of crude mortality rate were analyzed using Joinpoint Regression Program, Version 4.9.1.0—April 2022 (Statistical Methodology and Applications Branch, Surveillance Research Program, National Cancer Institute, USA) (https://surveillance.cancer.gov/joinpoint/). Microsoft Excel 2016 software (Microsoft, Redmond, WA, USA) was used to calculate distribution of causes of death and draw the histogram. SMR and AER were calculated by SEER*Stat software (version 8.4.0). SMR represents the observed-to-expected ratio. Observed deaths mean patient numbers die from a certain cause in the EOCRC population or a certain subgroup. Expected deaths were calculated using person-years multiplied by mortality rate of a certain cause in the general population provided by CDC WONDER. Person-years were calculated from the date of CRC diagnosis and ended at the date of death or the last follow-up, whichever came first. The AER was calculated as follows: (observed deaths − expected deaths)/10,000 person-years. The corresponding 95% confidence intervals (CIs) of the SMRs were calculated using Poisson regression models. Univariate and multivariate analyses were conducted utilizing Cox regression model. Hazard ratios (HRs) and the corresponding 95% CIs were reported. Two-tailed *p* < 0.05 was defined as statistically significant.

## Results

### Patient characteristics

Based on the above-mentioned criteria, 36,013 patients diagnosed with EOCRC in the SEER database were included in this study. The average follow-up time was 15.21 years (standard deviation, 0.08). Patient baseline characteristics are presented in **Table 1**. The majority of patients in this study aged at 40–49 years (71.1%), and were white (74.5%). Male patients were more than female patients (53.5% vs. 46.5%). Around half of the patients were diagnosed in 2004–2018 (49.1%). Most of the patients had low (I and II) grade tumor (59.2%), and most of the patients accepted surgery (88.1%) (**Table 1**). During follow-up time, 9,998 (27.7%) patients died of CRC, and 6,305 (17.5%) patients died of non-CRC causes.

**Table 1:** Baseline characteristics of early-onset colorectal cancer population.

Characteristics	No. of patients (%)	No. of CRC death (%)	No. of alive or non-CRC death (%)	*p-*value
All	36,013 (100)	9,998 (27.7)	26,015 (72.2)	
Age of diagnosis				< 0.001
20-29	2,226 (6.2)	720 (5.1)	1,506 (6.9)	
30-39	8,189 (22. 7)	3,093 (22.0)	5,096 (23.2)	
40-49	25,598 (71.1)	10,272 (72.9)	15,326 (69. 9)	
Sex				< 0.001
Male	19,251 (53.5)	7,771 (55.2)	11,480 (52.4)	
Female	16,762 (46.5)	6,314 (44.8)	10,448 (47. 6)	
Race				< 0.001
White	26,820 (74.5)	10,341 (73.4)	16,479 (75.2)	
Black	5,105 (14.2)	2,286 (16.2)	2,819 (12.9)	
Others^a^	4,088(11.4)	1,458 (10.4)	2,630 (12.0)	
Year of diagnosis				< 0.001
1975-1983	4,140 (11.5)	2,521 (17.9)	1,619 (7.4)	
1984-1993	5,745 (16.0)	3,065 (21.8)	2,680 (12.2)	
1994-2003	8,435 (23.4)	3,610 (25.6)	4,825 (22.0)	
2004-2018	17,693 (49.1)	4,889 (34.7)	12,804 (58.4)	
Grade^b^				< 0.001
Low (I+II)	21,334 (59. 2)	7,465 (53. 0)	13,869 (63. 2)	
High (III+IV)	6,187 (17. 2)	3,713 (26.4)	2,474 (11.3)	
Others^c^	206 (0.6)	62 (0.4)	144 (0.7)	
Unknown	8,286 (23. 0)	2,845 (20. 2)	5,441 (24.8)	
SEER stage				< 0.001
Localized	11,707 (32.5)	1,207 (8.6)	10,500 (47. 9)	
Regional	13,628 (37. 8)	4,757 (33.8)	8,871 (40.5)	
Distant	9,315 (25. 9)	7,519 (53.4)	1,796 (8. 2)	
Unknown	1,363 (3.8)	602 (4. 3)	761 (3. 5)	
Surgery				< 0.001
Yes	31,732 (88.1)	11,164 (79.3)	20,568 (93.8)	
No	4,023 (11.2)	2,782 (19.8)	1, 241 (5.7)	
Unknown	258 (0. 7)	139 (1.0)	119 (0.5)	

a Others include American Indian/Alaska Native and Asian/Pacific Islander.

b Low (grade I, well-differentiated and grade II, moderately differentiated) and high (grade III, poorly differentiated and grade IV, undifferentiated).

c Others include B-cell, pre-B-cell, B-precursor cell, and T-cell neoplasms.

### Mortality trends of EOCRC in the USA

**Figure 1:**
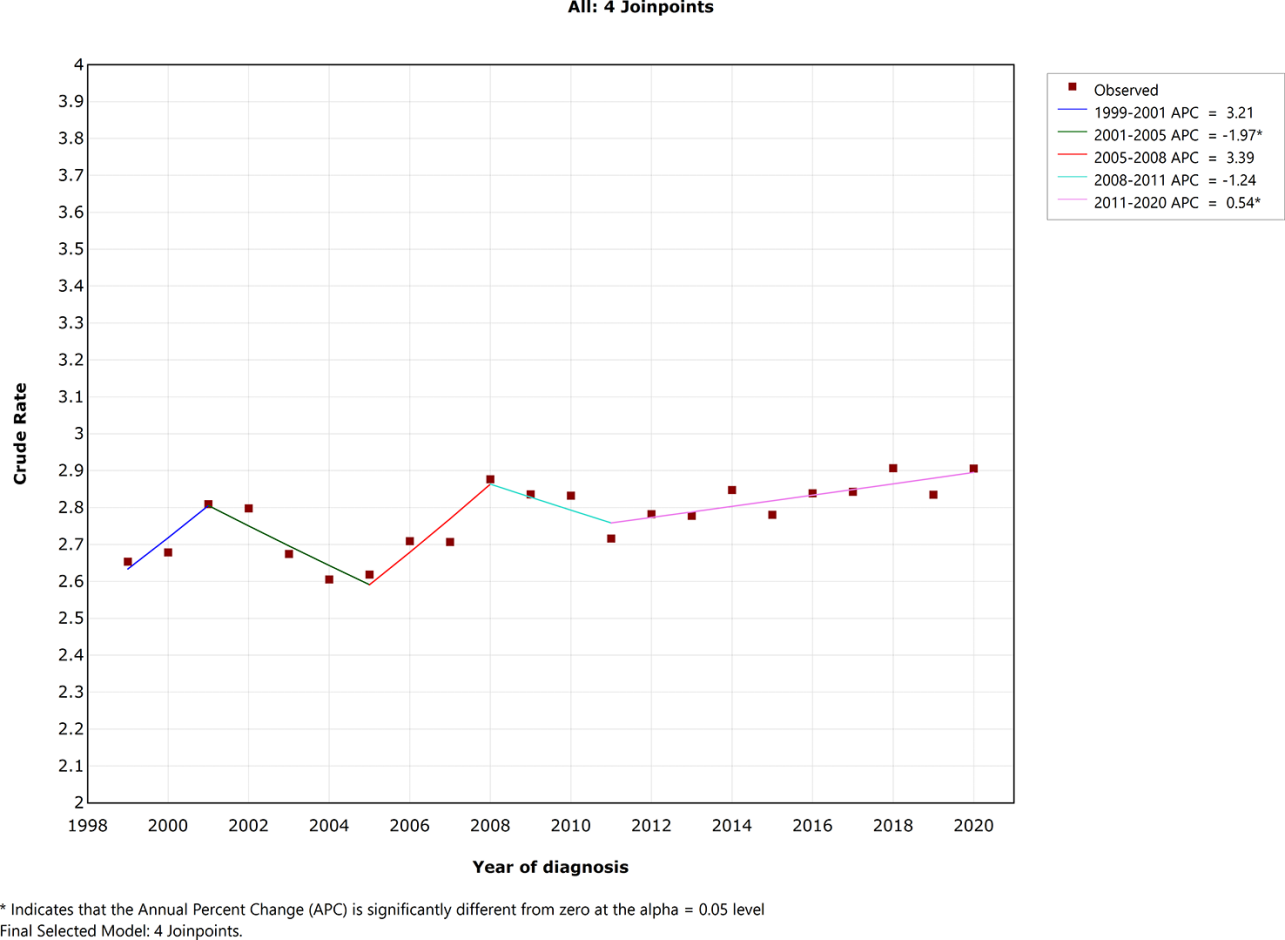
Trends of early-onset colorectal cancer death in the US population.

As presented in **Figure 1**, crude mortality rate was not stable during the last two decades. There were four joinpoints in the final selected model. Crude mortality rate decreased significantly during 2001–2005 [annual percentage change (APC) = −1.97; 95% CI, −3.8 to −0.1; *p* =0.040], whereas crude mortality rate increased significantly during 2011–2020 (APC = 0.54; 95% CI, 0.2–0.9; *p* = 0.006). Crude mortality rate change was not statistically significant in the year 1999–2001 (APC = 3.21; 95% CI, −0.6 to 7.2; *p* = 0.092), 2005–2008 (APC = 3.39; 95% CI, −0.4 to 7.3; *p* = 0.074), 2008–2011 (APC = −1.24; 95% CI, −4.8 to 2.4, *p* = 0.454).

### Distribution of causes of death at different follow-up time

**Figure 2:**
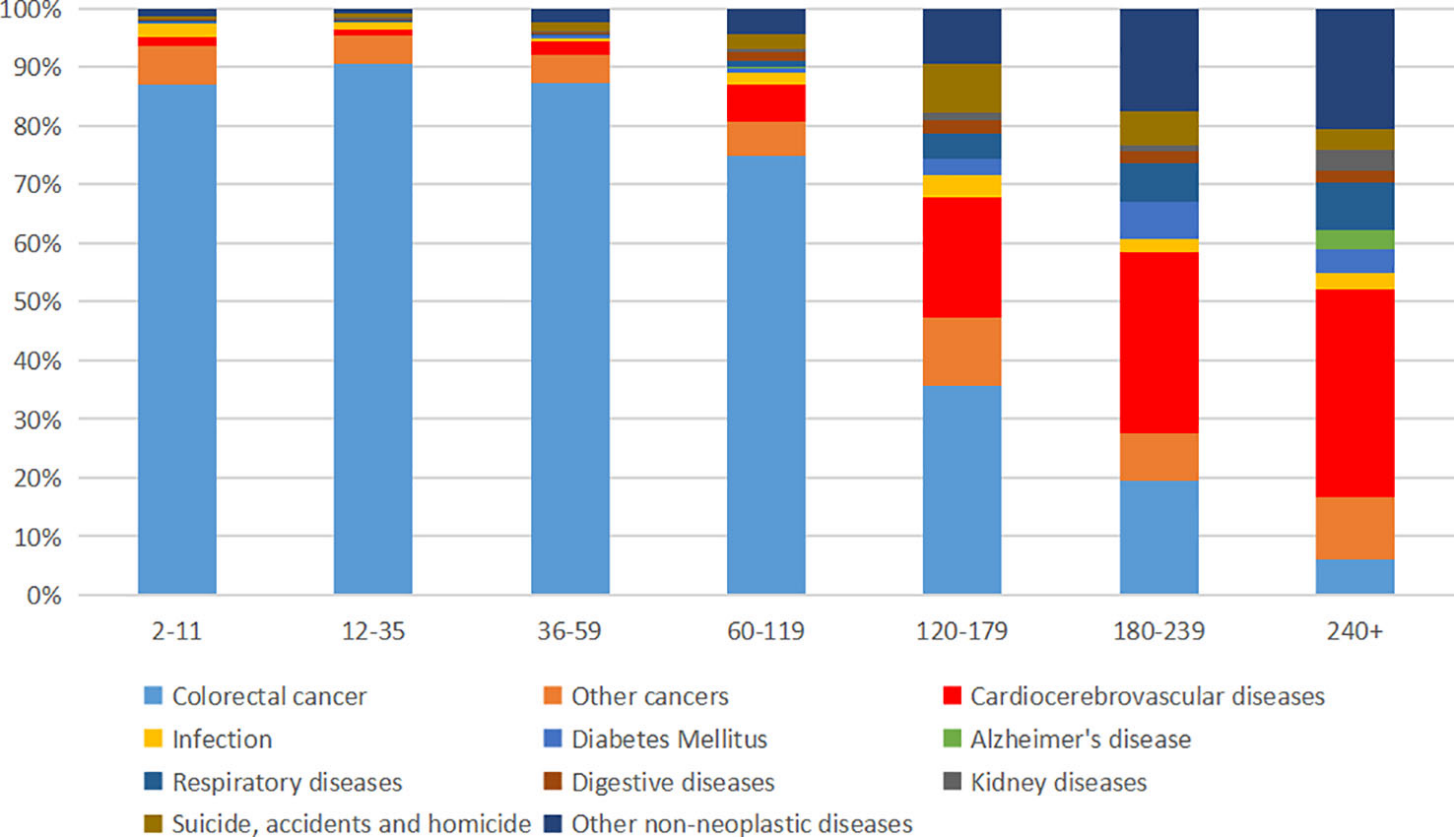
Proportion of death in early-onset colorectal cancer population at different follow-up times.

The proportion of each death cause is presented in **Figure 2** and **Supplementary Table S2**. CRC death was the major cause of death in 2–119 months after cancer diagnosis and accounted for a high proportion, with 87.08%, 90.69%, 87.24%, and 74.80% CRC-related death rate in 2–11, 12–35, 36–59, and 60–119 months, respectively (**Figure 1** and **Supplementary Table S2**). However, the proportion of CRC-related death reduced dramatically after 120 months, with 35.58%, 19.54%, and 5.93% CRC-related death rate in 120–179, 180–239, and 240+ months, respectively (**Figure 1** and **Supplementary Table S2**). Conversely, non-CRC death accounted for a high proportion of death after 120 months. Cardiocerebrovascular diseases (CVDs), other cancers, and respiratory diseases were the three most common non-CRC death causes. After 180 months, CVD exceeded CRC and became the leading cause of death in EOCRC population (**Figure 2**).

### SMRs and AERs for non-cancer causes of death

Detailed SMRs and AERs for each non-cancer cause of death at different follow-up times are presented in **Table 2**. It indicated a trend that the death risk of various causes was significantly higher than the general population in the relatively short period of follow-up, and the death risk of these causes was gradually approaching that of the general population over time.

**Table 2:** Standardized mortality ratios and absolute excess risks for causes of death on different follow-up time in early-onset colorectal cancer population.

Cause of death	2-11 months		12-35 months		36-59 months		60-119 months	
	SMR (95% CI)	AER	SMR (95% CI)	AER	SMR (95% CI)	AER	SMR (95% CI)	AER
**CVD**	2.95^#^ (2.22-3.84)	13.57	1.51^#^ (1.16-1.94)	4.20	1.36^#^ (1.01-1.80)	3.49	1.27^#^ (1.05 -1.52)	3.48
Diseases of heart	2.96^#^ (2.16-3.97)	11.13	1.49^#^ (1.11-1.97)	3.30	1.40^#^ (1.01 -1.89)	3.13	1.27^#^ (1.02 -1.54)	2.84
Hypertension without heart disease	2.58 (0.07-14.39)	0.23	1.13 (0.03-6.32)	0.02	1.23 (0.03-6.83)	0.05	0.46 (0.01 -2.58)	-0.16
Cerebrovascular diseases	3.10^#^ (1.34-6.11)	2.02	1.82 (0.87-3.34)	0.89	1.45 (0.58-3.00)	0.57	1.45 (0.84 -2.32)	0.74
Atherosclerosis	–	-0.02	–	-0.03	–	-0.03	–	-0.05
Aortic aneurysm and dissection	3.49 (0.09-19.46)	0.27	–	-0.12	–	-0.14	0.72 (0.02 -4.03)	-0.05
Other diseases of arteries, arterioles, capillaries	–	-0.06	2.84 (0.07-15.80)	0.13	–	-0.08	2.52 (0.30 -9.09)	0.17
**Infection**	11.26^#^ (5.15-21.38)	3.06	7.42^#^ (3.95-12.68)	2.24	3.81^#^ (1.40 -8.29)	1.16	2.48^#^ (1.19 -4.55)	0.84
Tuberculosis	–	-0.03	–	-0.03	–	-0.03	–	-0.03
Septicemia	12.54^#^ (5.73-23.81)	3.09	8.12^#^ (4.32-13.88)	2.27	4.10^#^ (1.51 -8.93)	1.19	2.62^#^ (1.26 -4.82)	0.87
**Diabetes mellitus**	4.64^#^ (2.12-8.80)	2.64	1.37 (0.50-2.99)	0.33	2.24^#^ (1.03 -4.26)	1.30	1.68 (1.00 -2.65)	1.03
**Alzheimer’s**	–	-0.01	–	-0.01	–	-0.01	4.09 (0.10 -22.76)	0.11
**Respiratory disease**	3.84^#^ (1.66-7.57)	2.21	3.36^#^ (1.92 -5.46)	2.24	0.90 (0.24 -2.30)	-0.12	1.45 (0.87 -2.27)	0.83
Pneumonia and influenza	5.95^#^ (2.18-12.94)	1.86	3.36^#^ (1.35-6.93)	0.98	1.70 (0.35 -4.96)	0.32	1.42 (0.52 -3.09)	0.25
Chronic obstructive pulmonary disease and allied cond	1.86 (0.23-6.74)	0.35	3.36^#^ (1.54-6.39)	1.26	0.37 (0.01 -2.07)	-0.44	1.47 (0.78 -2.51)	0.58
**Digestive disease**	2.30 (0.99-4.53)	1.69	1.79 (0.95-3.05)	1.14	1.27 (0.55 -2.51)	0.45	1.99^#^ (1.32 -2.88)	1.97
Stomach and duodenal ulcers	15.54^#^ (1.88-56.15)	0.70	–	-0.05	–	-0.06	3.65 (0.44 -13.18)	0.20
Chronic liver disease and cirrhosis	1.79 (0.66-3.90)	0.99	1.85 (0.99-3.17)	1.19	1.32 (0.57 -2.60)	0.51	1.93^#^ (1.26 -2.82)	1.76
**Kidney disease**	11.76^#^ (5.08-23.16)	2.73	6.01^#^ (2.75-11.41)	1.50	3.71^#^ (1.21 -8.67)	0.95	3.36^#^ (1.74 -5.87)	1.19
**Suicide, accidents, and homicide**	1.04 (0.61-1.64)	0.24	1.42^#^ (1.04-1.9)	2.72	1.42 (0.99 -1.98)	2.71	1.11 (0.82 -1.46)	0.68
Accidents and adverse effects	0.75 (0.32-1.48)	-0.99	1.09 (0.69-1.66)	0.38	1.42 (0.89 -2.15)	1.70	1.11 (0.76 -1.56)	0.43
Suicide and self-inflicted injury	1.83 (0.79-3.61)	1.36	2.53^#^ (1.57-3.87)	2.53	1.55 (0.74 -2.85)	0.93	1.15 (0.63 -1.93)	0.26
Homicide and legal intervention	0.86 (0.10-3.10)	-0.12	0.76 (0.16-2.23)	-0.19	1.13 (0.23 -3.29)	0.09	0.97 (0.26 -2.48)	-0.02
**Other cause of death**	5.17^#^ (3.87-6.76)	15.96	2.42^#^ (1.80-3.18)	5.96	2.92^#^ (2.18 -3.82)	8.93	2.06^#^ (1.65 -2.56)	6.11
**CVD**	1.14 (0.94-1.37)	2.80	1.10 (0.90-1.33)	2.99	1.00 (0.89-1.12)	0.03	1.17^#^ (1.09-1.26)	3.80
Diseases of heart	1.17 (0.94-1.43)	2.74	1.10 (0.88 -1.36)	2.33	1.01 (0.89-1.15)	0.72	1.19^#^ (1.10-1.28)	3.28
Hypertension without heart disease	2.04 (0.66-4.75)	0.52	1.14 (0.24-3.34)	0.11	0.88 (0.40-1.66)	-0.28	1.07 (0.66-1.64)	0.04
Cerebrovascular diseases	0.98 (0.51-1.71)	-0.05	1.17 (0.65-1.93)	0.65	1.00 (0.74-1.31)	-0.01	1.19 (0.99-1.42)	0.61
Atherosclerosis	–	-0.10	–	-0.16	0.45 (0.01-2.52)	-0.27	0.26 (0.01-1.42)	-0.09
Aortic aneurysm and dissection	–	-0.32	1.16 (0.14-4.19)	0.08	0.41 (0.05-1.47)	-0.64	0.54 (0.20-1.18)	-0.16
Other diseases of arteries, arterioles, capillaries	1.06 (0.03-5.88)	0.01	0.92 (0.02-5.14)	-0.03	1.61 (0.59-3.50)	0.50	1.49 (0.74-2.67)	0.12
**Infection**	1.13 (0.37-2.64)	0.12	0.64 (0.13-1.88)	-0.50	1.09 (0.64-1.75)	0.32	1.92^#^ (1.48-2.46)	0.96
Tuberculosis	5.59 (0.14-31.17)	0.17	–	-0.04	–	-0.06	0.87 (0.02-4.82)	–
Septicemia	0.94 (0.26-2.41)	-0.05	0.66 (0.14-1.93)	-0.46	1.11 (0.65-1.78)	0.38	1.96^#^ (1.50-2.51)	0.97
**Diabetes mellitus**	1.24 (0.69-2.04)	0.58	1.73^#^ (1.08-2.61)	2.76	0.96 (0.66-1.34)	-0.33	1.39^#^ (1.15-1.68)	1.00
**Alzheimer’s**	–	-0.11	–	-0.34	1.07 (0.71-1.55)	0.43	1.03 (0.69-1.48)	0.03
**Respiratory disease**	1.39 (0.89-2.07)	1.37	1.02 (0.64-1.55)	0.15	0.82 (0.64-1.03)	-3.44	1.09 (0.93-1.27)	0.45
Pneumonia and influenza	1.36 (0.50-2.95)	0.32	1.51 (0.61-3.12)	0.71	0.75 (0.41-1.26)	-1.03	1.33 (0.98-1.76)	0.39
Chronic obstructive pulmonary disease and allied cond	1.40 (0.83-2.21)	1.05	0.89 (0.50-1.47)	-0.56	0.84 (0.64-1.09)	-2.41	1.02 (0.84-1.22)	0.06
**Digestive disease**	1.00 (0.52-1.76)	0.01	0.77 (0.31-1.58)	-0.63	1.29 (0.77-2.04)	0.89	1.42^#^ (1.15-1.74)	0.89
Stomach and duodenal ulcers	–	-0.11	–	-0.15	0.78 (0.02-4.36)	-0.06	1.43 (0.46-3.34)	0.05
Chronic liver disease and cirrhosis	1.05 (0.54-1.84)	0.12	0.81 (0.33-1.67)	-0.48	1.34 (0.78-2.15)	0.95	1.42^#^ (1.14-1.75)	0.84
**Kidney disease**	1.68 (0.67-3.45)	0.57	0.84 (0.23-2.14)	-0.23	1.52^#^ (1.02-2.18)	2.17	2.10^#^ (1.65-2.64)	1.23
**Suicide, accidents, and homicide**	1.44^#^ (1.05-1.93)	2.73	1.00 (0.61-1.54)	-0.02	0.93 (0.63-1.31)	-0.57	1.20^#^ (1.05-1.36)	1.28
Accidents and adverse effects	1.20 (0.77-1.78)	0.81	1.10 (0.62-1.82)	0.43	0.98 (0.64-1.44)	-0.11	1.10 (0.93-1.29)	0.44
Suicide and self-inflicted injury	1.81^#^ (1.01-2.99)	1.36	0.94 (0.31-2.20)	-0.09	0.86 (0.32-1.88)	-0.21	1.53^#^ (1.21-1.90)	0.87
Homicide and legal intervention	2.23 (0.73-5.21)	0.56	–	-0.36	–	-0.25	0.96 (0.56-1.54)	-0.02
**Other cause of death**	1.31 (0.98-1.72)	2.45	1.65^#^ (1.27-2.12)	7.30	1.20^#^ (1.03-1.39)	6.38	1.69^#^ (1.55-1.84)	6.86

AER, absolute excess risk; CI, confidence interval; CVD, cardiocerebrovascular diseases; SMR, standardized mortality ratio.

^#^This value indicates statistical significance.

During 2- to 11-month follow-up, the EOCRC population had a high risk of death from kidney disease (SMR = 11.76; 95% CI, 5.08–23.16), infection (SMR = 11.26; 95% CI, 5.15–21.38), other causes of death (SMR = 5.17; 95% CI, 3.87–6.76), diabetes mellitus (SMR = 4.64; 95% CI, 2.12–8.80), respiratory disease (SMR = 3.84; 95% CI, 1.66–7.57), and CVD (SMR = 2.95; 95% CI, 2.22–3.84) (**Table 2**). Other causes of death and CVD had the highest AER (15.96 per 10,000 person-year and 13.57 per 10,000 person-year, respectively). SMRs and AERs of these causes declined within 10-year follow-up (**Table 2**). After 10 years, CVD, infection, and respiratory disease mortality risks were not significantly higher than the general population. During 120–179 months, suicide, accidents, and homicide risk was 1.44-fold higher than the general population (95% CI, 1.05–1.93). During 180–239 months, the mortality risk of diabetes mellitus was 1.73-fold higher than the general population (95% CI, 1.08–2.61) (**Table 2**).

For all follow-up time combination, patients had higher mortality risk of CVD (SMR = 1.17; 95% CI, 1.09–1.26), infection (SMR = 1.92; 95% CI, 1.48–2.46), diabetes mellitus (SMR = 1.39; 95% CI, 1.15–1.68), digestive disease (SMR = 1.42; 95% CI, 1.15–1.74), kidney disease (SMR = 2.10; 95% CI, 1.65–2.64), suicide, accidents, and homicide (SMR = 1.20; 95% CI, 1.05–1.36), and other cause of death (SMR = 1.69; 95% CI, 1.55–1.84) (**Table 2**). SMRs and AERs for each cause of death at different follow-up times in subgroups are presented in **Supplementary Tables S3–S9**.

### Prognostic factors for OS

As shown in **Table 3**, in univariate analysis, age at diagnosis, race, sex, year of diagnosis, grade, stage, and surgery were associated with overall survival. To avoid confounding factors, multivariate analysis was performed and further confirmed the above-mentioned factors as independent prognostic factors for OS. Compared with the younger patients, patients aged at 40–49 had higher death risk (HR = 1.23; 95% CI, 1.14–1.32, *p* < 0.001). Black patients had worse prognosis compared to white (HR = 1.29; 95% CI, 1.23–1.34, *p <*0.001). Female patients had better OS than male patients (HR = 0.88; 95% CI, 0.85–0.91, *p <*0.001) (**Table 3**). Diagnosed in more recent years indicated better OS. Patients with high-grade, regional or distant tumors, and no surgery evidence had worse OS.

**Table 3:** Univariate and multivariate analyses of overall survival.

Variables	Univariate analysis		Multivariate analysis	
	HR (95% CI)	*p*-value	HR (95% CI)	*p*-value
Age at diagnosis				
20-29 years	Reference		Reference	
30-39 years	1.15 (1.07-1.24)	<0.001	1.07 (0.99-1.15)	0.110
≥40 years	1.27 (1.18-1.36)	<0.001	1.23 (1.14-1.32)	<0.001
Race				
White	Reference		Reference	
Black	1.27 (1.22-1.32)	<0.001	1.29 (1.23-1.34)	<0.001
Others^a^	0.92 (0.88-0.97)	0.003	1.01 (0.96-1.06)	0.748
Sex				
Male	Reference		Reference	
Female	0.87 (0.85-0.90)	<0.001	0.88 (0.85-0.91)	<0.001
Year of diagnosis				
1975-1983	Reference		Reference	
1984-1993	0.76 (0.72-0.80)	<0.001	0.72 (0.69-0.75)	<0.001
1994-2003	0.57 (0.54-0.60)	<0.001	0.52 (0.50-0.55)	<0.001
2004-2018	0.43 (0.41-0.45)	<0.001	0.34 (0.32-0.35)	<0.001
Grade^b^				
Low	Reference		Reference	
High	1.97 (1.90-2.05)	<0.001	1.50 (1.45-1.56)	<0.001
Others^c^	0.94 (0.74-1.18)	0.592	0.68 (0.53-0.85)	0.001
Unknown	1.19 (1.15-1.24)	<0.001	1.00 (0.96-1.04)	0.980
Stage				
Localized	Reference		Reference	
Regional	2.63 (2.50-2.76)	<0.001	2.52 (2.40-2.65)	<0.001
Distant	10.52 (10.02-11.05)	<0.001	9.25 (8.79-9.74)	<0.001
Unknown	3.79 (3.49-4.12)	<0.001	2.54 (2.32-2.77)	<0.001
Surgery				
Yes	Reference		Reference	
No evidence	3.16 (3.04-3.30)	<0.001	2.11 (2.02-2.21)	<0.001
Unknown	2.14 (1.82-2.52)	<0.001	1.47 (1.25-1.73)	<0.001

a Others include American Indian/Alaska Native and Asian/Pacific Islander.

b Low (grade I, well-differentiated and grade II, moderately differentiated) and high (grade III, poorly differentiated and grade IV, undifferentiated).

c Others include B-cell, pre-B-cell, B-precursor cell, and T-cell neoplasms.

## Discussion

In the current long-term population-based study, we found that CRC death accounted for a high proportion of death within 10 years of cancer diagnosis, while non-CRC death, especially CVD death, was the major cause of death after 10 years. Compared to the general population, the EOCRC population had high risk of non-cancer death within 3 years after cancer diagnosis, but this disparity gradually diminished over time. Age at diagnosis, race, sex, year of diagnosis, grade, stage, and surgery were independent prognostic factors for OS. To the best of our knowledge, this is the first study focusing on cause of death in the EOCRC population.

EOCRC death was the main cause of death within 10-year follow-up, while non-EOCRC death became the leading cause of death after 10 years. Our results are similar to previous studies indicating that cancer-related death accounted for a high proportion of death in a relatively short period after diagnosis, whereas non-cancer death increased overtime and exceeded cancer-related death in long-term period (18–20). In CRC patients over 65 years old, Wang et al. also found that the proportions of CRC death were over 70% and 60% within 3 and 9 years after CRC diagnosis, respectively (9). Our results reflect a higher proportion of CRC death in the young population, with approximately 90% and 75% CRC death within 3- and 10-year follow-up. This may be explained by the relatively higher prevalence of comorbidities in older patients, and they are more likely to die from other diseases. Clinical guidelines of the American Society of Clinical Oncology (ASCO) emphasize the high risk of recurrence in 2–4 years after CRC diagnosis (21). In EOCRC population, more attention should be paid to CRC recurrence and metastasis within a longer period (10 years).

Non-CRC death is also worth noting in the EOCRC population. We observed a trend that the EOCRC population had high risk of non-CRC death compared to the age-matched general population, and the SMRs decrease overtime. A similar trend was also reflected in studies of CRC (22) or other solid cancers (23). A previous study conducted in the entire age CRC population (age ≤49 accounted for 0.996%) also showed relatively high non-CRC death risk in 2–11months, and SMRs in 12–119 months were lower; however, SMRs increased after 120 months (24). This difference may be attributed to the high prevalence of comorbidities in the AOCRC population (25) and long-term treatment side effect of anti-cancer treatment, whereas the long-term effect did not add excess death risk to the EOCRC population who had low prevalence of comorbidities. High non-CRC death risk in the short follow-up time may be explained by the side effect of cancer treatment. Further analysis using SMRs highlighting that not only should we pay attention to CRC death but also to non-CRC death within a 10-year follow-up.

Our results indicate that the risk of kidney disease and infection death in the EORCR population was over 10-fold to the age-matched general population in 2–11 months after cancer diagnosis. It was reported that acute kidney injury was common after colorectal cancer surgery, and it was correlated with mortality (26). Furthermore, chemotherapy was confirmed to have nephrotoxicity (27). Infection after surgery is also a frequent problem (28). Our results demonstrated increased respiratory disease death risk within 1-year follow-up in younger cancer population, which was consistent with a previous study (29). This is mainly caused by pneumonia and influenza in the present study. Similar to a study in CRC population (6), we also found increased diabetes mellitus death risk. Interestingly, we found that CVD death risk was highest in 2–11 months after diagnosis, and it decreased within 10 years. Our results are in line with a study by Gaitanidis et al., which showed high CVD death risk within 1 year after CRC diagnosis (30). A previous study including 563,298 CRC patients also found that CRC population had higher cerebrovascular-specific mortality risk than the general population (31). Our results could be explained by cardiotoxicity of aggressive chemotherapy in the EOCRC population. After 10 years, CVD death risk was not statistically different from the general population in the EOCRC population, which was distinct from AOCRC (24) and other cancers (32). We speculated that anti-cancer therapy had acute toxicity, but long-term CVD effect was not so obvious in young patients. Preventing these high-risk diseases helps to improve patients’ survival.

We confirmed that age at diagnosis, race, sex, year of diagnosis, grade, stage, and surgery were independent prognostic factors for OS in the EOCRC population, which was supported by previous studies (33–36). Worse prognosis in black population could be explained by poverty, less access to medical resource, and unfavorable tumor characteristics (34). Gender disparities in OS may be caused by the differences in life behaviors, genetics, and hormonal factors between men and women (37). With the advances of cancer detection and treatment, patients’ OS was significantly improved during the last four decades. Patients who had high stage or grade tumor had worse prognosis, and surgery was an effective treatment. These independent prognostic factors assist to identify high-risk patients.

The current study has some limitations. First, this is a retrospective study, and some unknown information may cause bias. Second, the population in the study span a long time and disease classification, treatment, and survival care change during this period, which may also affect the results. Third, limited data in the SEER database do not allow us to further analyze reasons behind each cause of death. However, the main purpose of the study is to describe causes of death in the EOCRC population. Fourth, the newest histological and molecular factors including tumor budding, microsatellite instability, epithelial–mesenchymal transition, and molecular classification also had prognostic impact (38). However, data from the SEER database lack this information, which limited us to further analyze the prognostic effect of the newest histological and molecular factors. Finally, our results should be interpreted with caution when they are extrapolated to population outside the US because these results are based on the US population. Despite these limitations, the present study is the first to focus on causes of death in a large-scale EOCRC population with long-term follow-up.

## Conclusions

In conclusion, we found that death from CRC accounted for a high proportion within a 10-year follow-up, and non-CRC death exceeded it after 10 years. Compared to the general population, patients had high risk of non-cancer death, especially within the first year after cancer diagnosis. Non-CRC death cannot be ignored after EOCEC diagnosis. Monitoring and preventing these high risk causes help to improve patients’ survival in the US.

## Data availability statement

The raw data supporting the conclusions of this article will be made available by the authors, without undue reservation.

## Ethics statement

Patients’ informed consents and Institutional Review Board (IRB) approval are not required, since these data are publicly available in the database.

## Author contributions

YRC: conceptualization, data curation, roles/writing—original draft, investigation, and software. LH: data curation, writing—original draft, resources, and validation. XL: data curation, visualization, and investigation. YT: formal analysis, methodology, and software; GL: writing—original draft and validation. YJC: writing—data curation and visualization. CW: software and data curation. QL: conceptualization, project administration, supervision, and writing—review and editing. XX: conceptualization, funding acquisition, writing—review and editing, and supervision. All authors contributed to the article and approved the submitted version.
