# Supplementary material for: Causes of death among early-onset colorectal cancer population in the United States: a large population-based study
Source: Front Oncol. 2023 Apr 24;13:1094493. doi: 10.3389/fonc.2023.1094493 (PMC10166590; doi:10.3389/fonc.2023.1094493)
Supplement: Supplementary file 1 [file DataSheet_1.docx]

**Supplementary table legends**

**Table S1**. Detailed causes of death classification.

Abbreviation: ICD = International Classification of Diseases.

**Table S2.** Number and proportion of death from each cause at different follow-up time.

**Table S3.** SMR and AER in different age groups.

**Table S4.** SMR and AER in different gender groups.

**Table S5.** SMR and AER in different grade groups.

**Table S6.** SMR and AER in different race groups.

**Table S7.** SMR and AER in different SEER stage groups.

**Table S8.** SMR and AER in different treatment groups.

**Table S9.** SMR and AER in different year of diagnosis groups.

**Supplementary figure legends**

**Figure S1**. Age distribution in early-onset colorectal cancer population.

**Table S1.** Detailed causes of death classification.

| **Causes of death** | **Detailed disease classification** |
| --- | --- |
| Colorectal cancer | Colon cancer; rectal cancer |
| Other cancers | In situ, benign or unknown behavior neoplasm; Melanoma of the Skin; Mesothelioma (ICD-10 only); Miscellaneous Malignant Cancer; Non-Melanoma Skin; Soft Tissue including Heart; Brain and Other Nervous System; Lung and Bronchus; Colon excluding Rectum; Esophagus; Intrahepatic Bile Duct; Liver; Pancreas; Rectum and Rectosigmoid Junction; Salivary Gland; Stomach; Kidney and Renal Pelvis; Prostate; Ureter; Acute Monocytic Leukemia; Acute Myeloid Leukemia; Bones and Joints; Chronic Lymphocytic Leukemia; Non-Hodgkin Lymphoma; Testis |
| Cardiovascular | Aortic Aneurysm and Dissection; Atherosclerosis; Cerebrovascular Diseases; Diseases of Heart; Hypertension without Heart Disease; Other Diseases of Arteries, Arterioles, Capillaries |
| Infection | Other Infectious and Parasitic Diseases including HIV; Septicemia; Tuberculosis; Syphilis |
| Endocrine | Diabetes Mellitus |
| Nervous | Alzheimer’s (ICD-9 and 10 only) |
| Respiratory | Chronic Obstructive Pulmonary Disease and Allied Cond; Pneumonia and Influenza |
| Digestive | Chronic Liver Disease and Cirrhosis; Stomach and Duodenal Ulcers |
| Genitourinary | Nephritis, Nephrotic Syndrome and Nephrosis |
| Suicide, accidents and homicide | Accidents and Adverse Effects; Suicide and Self-Inflicted Injury; Homicide and Legal Intervention |
| Other non-neoplastic diseases | Certain Conditions Originating in Perinatal Period; Complications of Pregnancy, Childbirth, Puerperium; Congenital Anomalies; Other Cause of Death; Symptoms, Signs and Ill-Defined Conditions |

Abbreviation: ICD = international Classification of diseases

**Table S2.** Number and proportion of death from each cause at different follow-up time.

| Causes of death | Follow-up time (months) | | | | | | | | | | | | | |
| --- | --- | --- | --- | --- | --- | --- | --- | --- | --- | --- | --- | --- | --- | --- |
|  |  | 2-11 |  | 12-35 |  | 36-59 |  | 60-119 |  | 120-179 |  | 180-239 |  | 240+ |
| Colorectal cancer |  | 3162 (87.08%) |  | 5259 (90.69%) |  | 2017 (87.24%) |  | 1425 (74.80%) |  | 190 (35.58%) |  | 68 (19.54%) |  | 51 (5.93%) |
| Other cancers |  | 239 (6.58%) |  | 272 (4.69%) |  | 112 (4.84%) |  | 114 (5.98%) |  | 62 (11.61%) |  | 28 (8.05%) |  | 92 (10.70%) |
| Cardiocerebrovascular diseases |  | 49 (1.35%) |  | 62 (1.07%) |  | 50 (2.16%) |  | 118 (6.19%) |  | 110 (20.60%) |  | 107 (30.75%) |  | 305 (35.47%) |
| Infection |  | 86 (2.37%) |  | 67 (1.16%) |  | 17 (0.74%) |  | 38 (1.99%) |  | 20 (3.75%) |  | 8 (2.30%) |  | 25 (2.91%) |
| Diabetes Mellitus |  | 9 (0.25%) |  | 6 (0.10%) |  | 9 (0.39%) |  | 18 (0.94%) |  | 15 (2.81%) |  | 22 (6.32%) |  | 33 (3.84%) |
| Alzheimer's disease |  | 0 (0.00%) |  | 0 (0.00%) |  | 0 (0.00%) |  | 1 (0.05%) |  | 0 (0.00%) |  | 0 (0.00%) |  | 28 (3.26%) |
| Respiratory diseases |  | 8 (0.22%) |  | 15 (0.26%) |  | 4 (0.17%) |  | 20 (1.05%) |  | 23 (4.31%) |  | 23 (6.61%) |  | 71 (8.26%) |
| Digestive diseases |  | 7 (0.19%) |  | 13 (0.22%) |  | 8 (0.35%) |  | 28 (1.47%) |  | 12 (2.25%) |  | 7 (2.01%) |  | 18 (2.09%) |
| Kidney diseases |  | 5 (0.14%) |  | 9 (0.16%) |  | 5 (0.22%) |  | 12 (0.63%) |  | 7 (1.31%) |  | 4 (1.15%) |  | 29 (3.37%) |
| Suicide, accidents and homicide |  | 15 (0.41%) |  | 46 (0.79%) |  | 35 (1.51%) |  | 49 (2.57%) |  | 44 (8.24%) |  | 20 (5.75%) |  | 32 (3.72%) |
| Other non-neoplastic diseases |  | 51 (1.40%) |  | 50(0.86%) |  | 55 (2.38%) |  | 82 (4.30%) |  | 51 (9.55%) |  | 61 (17.53%) |  | 176 (20.47%) |


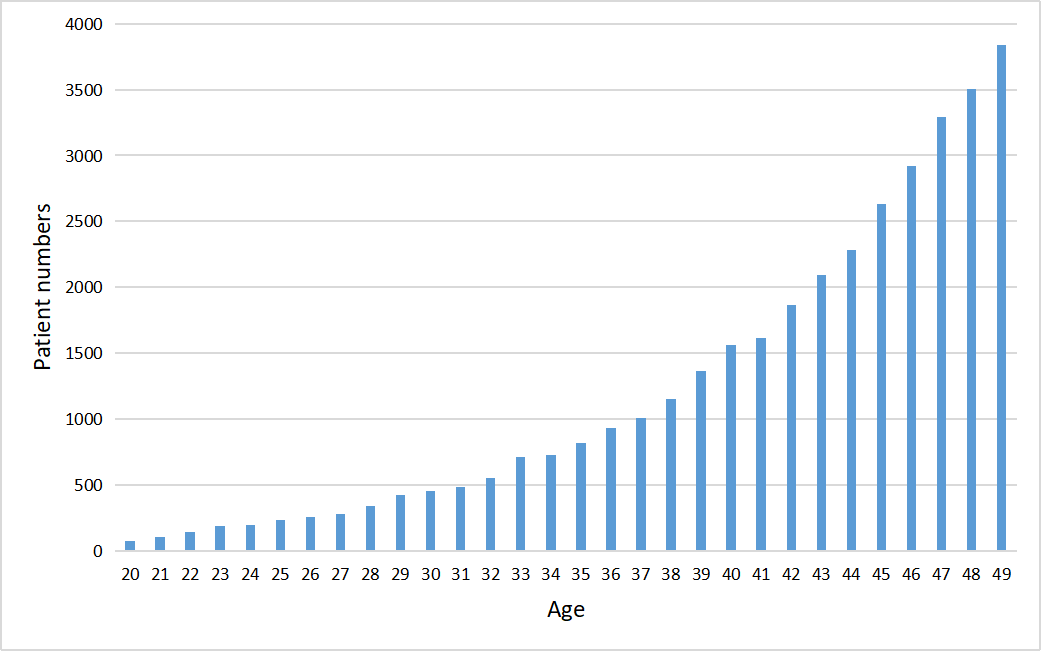


**Figure S1.** Age distribution in early-onset colorectal cancer population.
